# Differential Expression of AMPA Subunits Induced by NMDA Intrahippocampal Injection in Rats

**DOI:** 10.3389/fnins.2016.00032

**Published:** 2016-02-15

**Authors:** Helene A. Fachim, Adriana C. Pereira, Melina M. Iyomasa-Pilon, Maria L. N. M. Rosa

**Affiliations:** ^1^Department of Biology, Faculty of Philosophy, Sciences and Letters of Ribeirao Preto, University of Sao PauloRibeirão Preto, Brazil; ^2^Institute of Neuroscience and BehaviorRibeirão Preto, Brazil; ^3^Faculty of Medicine of CatanduvaCatanduva, Brazil; ^4^Barretos School of Health Sciences, Faculdade de Ciências da Saúde de Barretos Dr. Paulo Prata (FACISB)Barretos, Brazil

**Keywords:** AMPA receptors, hippocampus, neurodegenerative disorders, NMDA, excitotoxicity, immunohistochemistry, Morris water maze

## Abstract

Glutamate is involved in excitotoxic mechanisms by interacting with different receptors. Such interactions result in neuronal death associated with several neurodegenerative disorders of the central nervous system (CNS). The aim of this work was to study the time course of changes in the expression of GluR1 and GluR2 subunits of glutamate amino-acid-3-hydroxy-5-methyl-isoxazol-4-propionic acid (AMPA) receptors in rat hippocampus induced by NMDA intrahippocampal injection. Rats were submitted to stereotaxic surgery for NMDA or saline (control) microinjection into dorsal hippocampus and the parameters were evaluated 24 h, 1, 2, and 4 weeks after injection. The extension and efficacy of the NMDA-induced injury were evaluated by Morris water maze (MWM) behavioral test and Nissl staining. The expression of GluR1 and GluR2 receptors, glial fibrillary acidic protein (GFAP), and neuronal marker (NeuN) was analyzed by immunohistochemistry. It was observed the impairment of learning and memory functions, loss of neuronal cells, and glial proliferation in CA1 area of NMDA compared with control groups, confirming the injury efficacy. In addition, NMDA injection induced distinct changes in GluR1 and GluR2 expression over the time. In conclusion, such changes may be related to the complex mechanism triggered in response to NMDA injection resulting in a local injury and in the activation of neuronal plasticity.

## Introduction

Glutamate is the main and most abundant excitatory neurotransmitter in the Central Nervous System (CNS) with key role in several physiological functions. However, excessive concentrations of glutamate result in overstimulation of the ionotropic receptors leading to increased Ca^2+^ influx into the neurons. High Ca^2+^ levels trigger an intracellular mechanism known as excitotoxicity that induces neuronal death. This cascade of events has been reported in most neurodegenerative disorders of the CNS (Ozawa et al., 1998; Kim and Frank, 2009).

The ionotropic glutamate receptors (iGluRs) are a complex of transmembrane proteins assemblies that reflect the receptor permeability to the influx of ions (Hollmann and Heinemann, 1994; Rosa, 2006). These receptors are classified according to their agonists N-methyl-D-aspartate (NMDA), amino-acid-3-hydroxy-5-methyl-isoxazol-4-propionic acid (AMPA), and kainate (KA). Specifically, the AMPA receptors are formed by the assembly of four different protein subunits, known as GluR1-4, derived from a single gene. GluR1, 3, and 4 have different permeabilities to Ca^2+^ while GluR2 is totally impermeable. The presence of at least one GluR2 in the receptor composition is enough to reduce the Ca^2+^ permeability (Meldrum, 2000). In addition, the alternative splicing of genes encoding the subunits originates two isoforms of each, flip and flop, with distinct physical and chemical properties. The distribution of these subunits and isoforms is different considering the brain region and the stage of development (Sommer et al., 1990; Hollmann and Heinemann, 1994; Pires et al., 2000).

Some evidences have indicated the involvement of glutamate by interacting with different receptors in the mechanisms underlying the CNS development, including cell proliferation and migration, morphological changes of neurites, neuronal differentiation, and neurogenesis (Pires et al., 2006; Ritter, 2011). Considering the AMPA receptors, studies have demonstrated that they are differentially regulated through the expression of specific subunits resulting in distinct Ca^2+^ influx that affects neuronal functions such as synaptic plasticity and neuronal survival or excitotoxicity (Pellegrini-Giampietro, 2003; Pires et al., 2006). The ion exchange through neuronal membrane induces activation of voltage-dependent NMDA receptors in the membrane and cellular changes that trigger intracellular responses such as activation of second messengers (Malinow and Malenka, 2002; Sheng and Lee, 2003).

Overstimulation of NMDA receptors also induces marked increase of Ca^2+^ influx as all subunits of these receptors are permeable to this ion. Several studies have shown the involvement of Ca^2+^ influx through these receptors in mechanisms underlying short- or long-term alterations in the CNS such as long-term potentiation (LTP) that is essential for memory formation and a cellular model of synaptic plasticity (Prybylowski and Wenthold, 2004). In addition to NMDA, the presence of GluR1 in AMPA receptors contributes to synaptic potentiation during LTP (Rumpel et al., 2005) as well as receptors deficient in GluR2 contribute to enhanced LTP, suggesting an important regulatory mechanism associated with the composition of AMPA receptors (Jia et al., 1996).

NMDA intrahippocampal injection induces substantial loss of hippocampal tissue and gliosis (Bardgett et al., 2003) and has been largely used as an experimental model of neurodegenerative processes such as ischemia, Alzheimer's and Parkinson's disease, multiple and amyotrophic lateral sclerosis, epilepsy, and aging (Bowie, 2008). It has been demonstrated that this injury induces impairment of spatial memory in rats (Bardgett et al., 2003, 2006) as mammalian hippocampus is involved in mechanisms underlying the acquisition of new information, learning, and memory (Bertoglio et al., 2006).

Although the involvement of AMPA receptors in neurodegenerative processes is already known (Ozawa et al., 1998), changes in the expression of specific subunits after injury have not been demonstrated in a time course way. Thus, the aim of this study was to analyze by immunohistochemistry GluR1 and GluR2 expression in hippocampus (CA1, CA3, and hilus of dentate gyrus at different time points after local microinjection of NMDA in rats. The lesion efficacy was evaluated by behavioral test using the Morris water maze (MWM), one of the most widely spatial memory test used in rodents (Talpos et al., 2008). The histological alterations related to the loss of neurons and glial proliferation were evaluated by Nissl staining and immunohistochemistry.

## Materials and methods

### Animals and housing

Forty eight male Wistar rats (220–250 g) were allocated in pairs in a temperature-controlled room (23 ± 1°C) and a 12:12-h light:dark cycle (lights on at 7 a.m.) with food and water *ad libitum*. Animal handling and all experiments were performed according to the Brazilian Society of Neuroscience and Behavior guidelines for care and use of laboratory animals, and all efforts were made to minimize animal suffering. This study was approved by the Ethics Committee for Care and Use of Laboratory Animals of the University of Sao Paulo, campus Ribeirao Preto (CEUA # 10.1.619.53.3).

### Surgery and NMDA intrahippocampal injection

Eight experimental groups were used (*n* = 6/each): Control and NMDA (analyzed 24 h, 1, 2, and 4 weeks after either saline or NMDA injection, respectively). Fifteen minutes before the surgical procedure the animals received atropine sulfate (0.1 mL, 0.5 mg/mL) and then were anesthetized with ketamine hydrochloride (60 mg/kg; Agener Union) plus xylazine (8 mg/kg; Cali). They were fixed in a stereotaxic device (Stoelting-Standard) and received a local injection of lidocaine (2%, S.S. White 100). After 30 s an incision was made on the top of the head in order to expose the skull. Based on the bregma point (the junction point of the sagittal and coronal sutures of the skull) the following coordinates were used to reach the dorsal hippocampus, according to Paxinos and Watson (1986): AP: −3.8, ML: ± 1.4, DV: −2.0. Then, the animals received a bilateral injection of NMDA (0.2 mL, 12 mg/mL, Sigma) and intramuscular injections of diazepam (1 mg/kg, Cristalia) to prevent the occurrence of seizures, and antibiotics (penicillin, streptomycin, and dihydrostreptomycin) (50 mg/kg, Fort Dodge). The incisions were sutured and the animals were left undisturbed until behavioral test or histological procedures.

### Behavioral test (MWM)

Two and 4 weeks after either NMDA or saline injection, the animals (*n* = 6/group) were tested in the MWM (Morris, 1984), using a circular polyethylene pool (1.40 cm diameter and 50 cm deep) filled with water (23°C) containing 2 L of milk to prevent the animals of seeing the platform. A white platform (9 cm diameter) was allocated on the southeast quadrant and visual cues (geometric figures: square, triangle, circle, and star) were placed on the walls of the room. The experimental protocol consisted of 24 training sessions (six sessions/day for four consecutive days) and one test session (*probe trial*, day 5). Each session lasted until either the animal find the platform or the cutting time of 90 s, when the animal was removed. In this case, the animal was manually driven to the platform, kept on that for 30 s and again placed in water. In the training sessions the escape latencies to find the platform were recorded. At day 5, the platform was removed to perform the *probe trial* that consisted of recording the swimming time in each quadrant during 60 s in the maze. The time spent in the quadrant where the platform used to be was taken as an index of memory. The test evaluated the ability of acquisition (training sessions with the platform) and retention (test session without the platform) of the spatial information. One day and 1 week groups were not included in this behavioral test as the rats must be recovered from the surgery for at least 1 week. Thus, they can not be submitted to the test considering 1 week of training needed (1–4 days) before the *probe trial* (day 5).

### Histological procedures

After the behavioral test, all animals were anesthetized (urethane, 25%, Sigma) and killed by perfusion with intracardiac infusion of 0.1 M phosphate buffered saline (PBS), pH 7.4, followed by 4% paraformaldehyde in PBS. The brains were promptly removed and soaked in the same fixative solution for 2 h (4°C) and then cryoprotected by soaking in 30% sucrose in PBS for 48–72 h (4°C). Brains were frozen in isopentane (Sigma) cooled in dry ice (−40°C) and stored at −70°C until sectioning. Twenty-four hours before sectioning the brains were transferred to a −20°C freezer. Selection of anatomical levels for sectioning was conducted based on illustrations from Paxinos and Watson (1986). Transverse sections (30 μm) containing hippocampus were obtained in a cryostat (−20°C, Leica) and processed for Nissl staining (3 sections/animal from control and NMDA 2 and 4 weeks) or immunohistochemistry (3 sections/animal).

### Immunohistochemistry

For immunohistochemistry revealed with chromogen 3,3-diaminobenzidine (DAB. Sigma), hippocampal sections of all groups were successively washed and incubated at room temperature with the anti-GluR1 (1:500, Chemicon) or anti-GluR2 (1:400, Millipore) primary antibodies. After 18 h, they were washed in PBS and sequentially incubated with a biotinylated secondary antibody (1:250, Dako) for 90 min. The sections were then processed by the avidin-biotin immunoperoxidase method (Vectastain ABC kit, Vector Laboratory). GluR1 or GluR2 immunoreactivity was revealed by the addition of DAB and hydrogen peroxide (Merck). The sections were mounted on gelatin-coated slides, dehydrated, and cover slipped for microscopic analysis. The sections were observed and the subfields of the hippocampus (CA1, CA3, and hilus) were captured using a light microscope coupled with a digital camera (DFC300 FX, Leica) for further quantification using the image analysis software QWin Plus (Leica). GluR1- and GluR2-positive neurons were visualized as brown in color. Counting of GluR1- and GluR2-immunopositive cells (IC) was performed in the entire field of view at 200x magnification (i.e., 10x eyepiece and 20x plan objective), corresponding to a fixed area of 566 × 424 μm (0.24 mm^2^) for each hippocampal region. Images of each region in the magnification used for quantifying the positive cells are showed in the Supplementary Material (GluR1, Figure S1 and GluR2, Figure S2). One whole field per region was counted in three sections from each animal, bilaterally (6 values/region/rat) and the values used to calculate the average expressions. The results were expressed as the number of GluR1- and GluR2-IC/0.1 mm^2^. As the NMDA injection was made into the CA1, the IC of this region were counted in the lesion adjacent area. Due to the high density of GluR1- and GluR2-IC in the layers of neurons in CA1 and CA3, the expression of both subunits in these areas was also measured by densitometry. The images were captured and transformed in black and white and the optical density (OD) was measured in gray scale using the Image J software. The analyzed areas were measured on right and left hemispheres in all three sections of each animal and the readings used to calculate the average expression.

For immunofluorescence, tissue sections of the 2 weeks control and 1 and 4 weeks NMDA groups (*n* = 2/each) were used. Eighteen hours before the experiment, the brain tissues were moved from anti-freeze solution to phosphate buffer (PB) 0.1 M with Triton X-100 0.15%. Tissue sections were successively washed and incubated at room temperature with primary antibodies mouse anti-NeuN (1:1000, Millipore) and mouse anti-GFAP (1:1000, Sigma) to detect neurons and glial cells, respectively. After 60 min all sections were washed in PB 0.1 M and sequentially incubated with the secondary antibodies (Alexa Fluor 594-red or Alexa Fluor 488-green). After 50 min, all sections were washed in PB 0.1 M and incubated with DAPI for 5 min to label cell nuclei. The sections were washed again in PB 0.1 M and deionized water and mounted on non-gelatin-coated slides with anti-fade and cover slipped for analysis using fluorescence microscope outfitted with an Axiocam MRm digital camera (Carl Zeiss).

### Statistical analysis

The statistical analyses were preformed using Graph Prism software (version 4.0, GraphPad Software). The data from MWM test were compared by repeated measures either two-way or one-way ANOVA followed by Bonferroni test. For Nissl and imunnohistochemistry experiments, the data were compared by one-way ANOVA followed by Newman–Keuls test. The significance level was set at *p* ≤ 0.05.

## Results

### Behavioral test (MWM)

**Figure 1:**
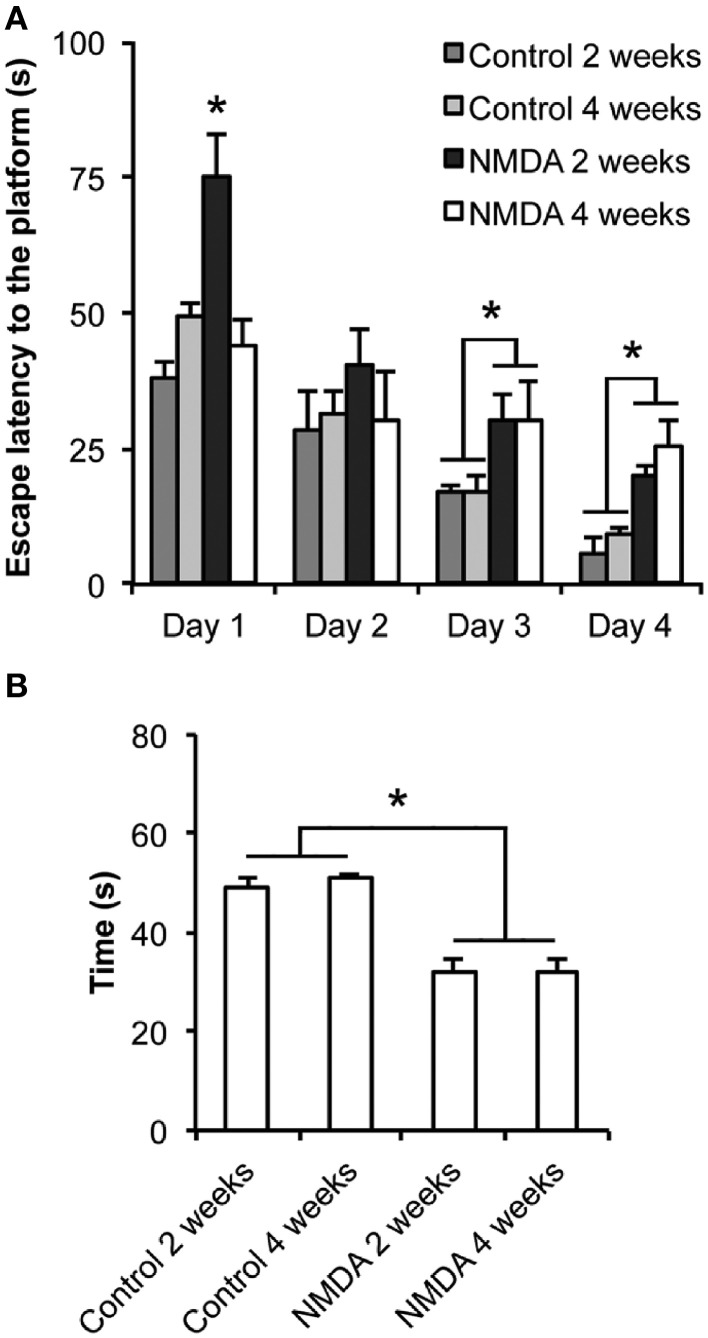
**Effect of intrahippocampal NMDA in MWM acquisition task 2 and 4 weeks after injection. (A)** Escape latency to find the platform during the four training sessions. **(B)** Time spent in the target quadrant during the *probe trial* (without the platform). Data represent mean ± SEM (*n* = 6/group). ^*^p < 0.01 (Repeated measures two-way **(A)** or one-way **(B)** ANOVA followed by Bonferroni test).

There were significant differences in the learning (Figure 1A) and memory (Figure 1B) functions between control and NMDA injected groups. The escape latency to find the platform was progressively reduced from day 1 to day 4 of training for all groups [*F*_(3, 60)_ = 2.265; *p* < 0.0001; Figure 1A]. At day 1, animals tested 2 weeks after NMDA injection showed an escape latency higher than both controls and animals tested 4 weeks after NMDA injection [*F*_(3, 20)_ = 14.14; *p* < 0.0001]. However, no significant difference was observed between NMDA tested at 4 weeks and control groups. At day 2, no difference in the escape latency was observed between all groups. In contrast, at day 3 the NMDA groups tested at 2 and 4 weeks after injection showed an escape latency 130 and 76% higher than controls, respectively [*F*_(3, 20)_ = 22.30; *p* < 0.0001]. Similarly, at day 4, the NMDA groups showed an escape latency 198 and 177% higher than controls [*F*_(3, 20)_ = 11.87; *p* < 0.01]. In the *probe trial* carried out at day 5, both NMDA injected groups spent 40% less time swimming in the target quadrant than controls [*F*_(3, 20)_ = 25.55; *p* < 0.0001; Figure 1B]. No difference was detected between both NMDA injected groups [*F*_(3, 20)_ = 1.172; *p* = 0.978].

### Hippocampus injury induced by NMDA injection

**Figure 2:**
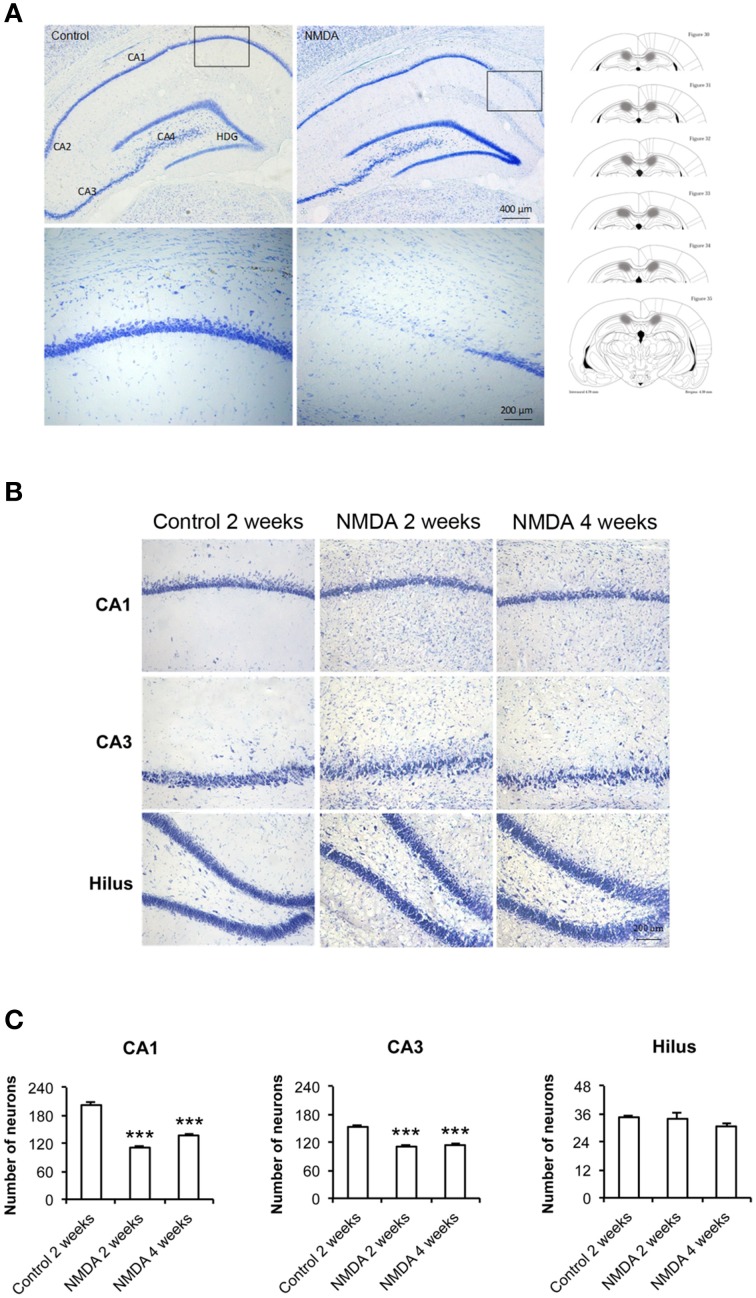
**(A)** Dorsal hippocampus of the control and NMDA groups 2 weeks after injection. Lower (top) and higher magnification of the bounded areas (bottom). Plates used for neuron quantification (right) showing the injured area (hachured) (modified from Paxinos and Watson, 1986). **(B)** CA1, CA3, and hilus of the control, after 2 weeks, and NMDA after 2 and 4 weeks. **(C)** Number of neurons in CA1, CA3, and hilus of the control, after 2 weeks, and NMDA after 2 and 4 weeks. Data represent mean ± SEM of the number of cells quantified bilaterally in three sections/rat (*n* = 6/group). Nissl staining. ^***^p < 0.001 (one-way ANOVA followed by Newman–Keuls test).

At lower magnification, it was possible to observe the whole hippocampus and to identify its specific areas, CA1, CA2, CA3, and hilus, in control and NMDA groups 2 weeks after injection (Figure 2A). It was noticed the loss of neurons in the CA1 region of the NMDA injected compared with control group (Figure 2A, bounded areas and higher magnification) indicating the lesion effectiveness. A higher amount of glial cells and a reduction in neuronal layer thickness was observed in NMDA groups, 2 and 4 weeks after injection, compared with control group 2 weeks after injection (Figure 2B). A significant neuronal loss was observed in CA1 and CA3 areas of the NMDA groups, 2 and 4 weeks after injection, compared with control group 2 weeks after injection (Figure 2C). The reduction was 50% after 2 weeks and 31% after 4 weeks [*F*_(2, 17)_ = 111.2; *p* < 0.0001] in CA1 and 24% after 2 weeks and 27% after 4 weeks in CA3 [*F*_(2, 17)_ = 54.96; *p* < 0.0001]. In the hilus, no significant difference was observed [*F*_(2, 17)_ = 1.393; *p* = 0.279].

### GluR1 expression in hippocampus

**Figure 3:**
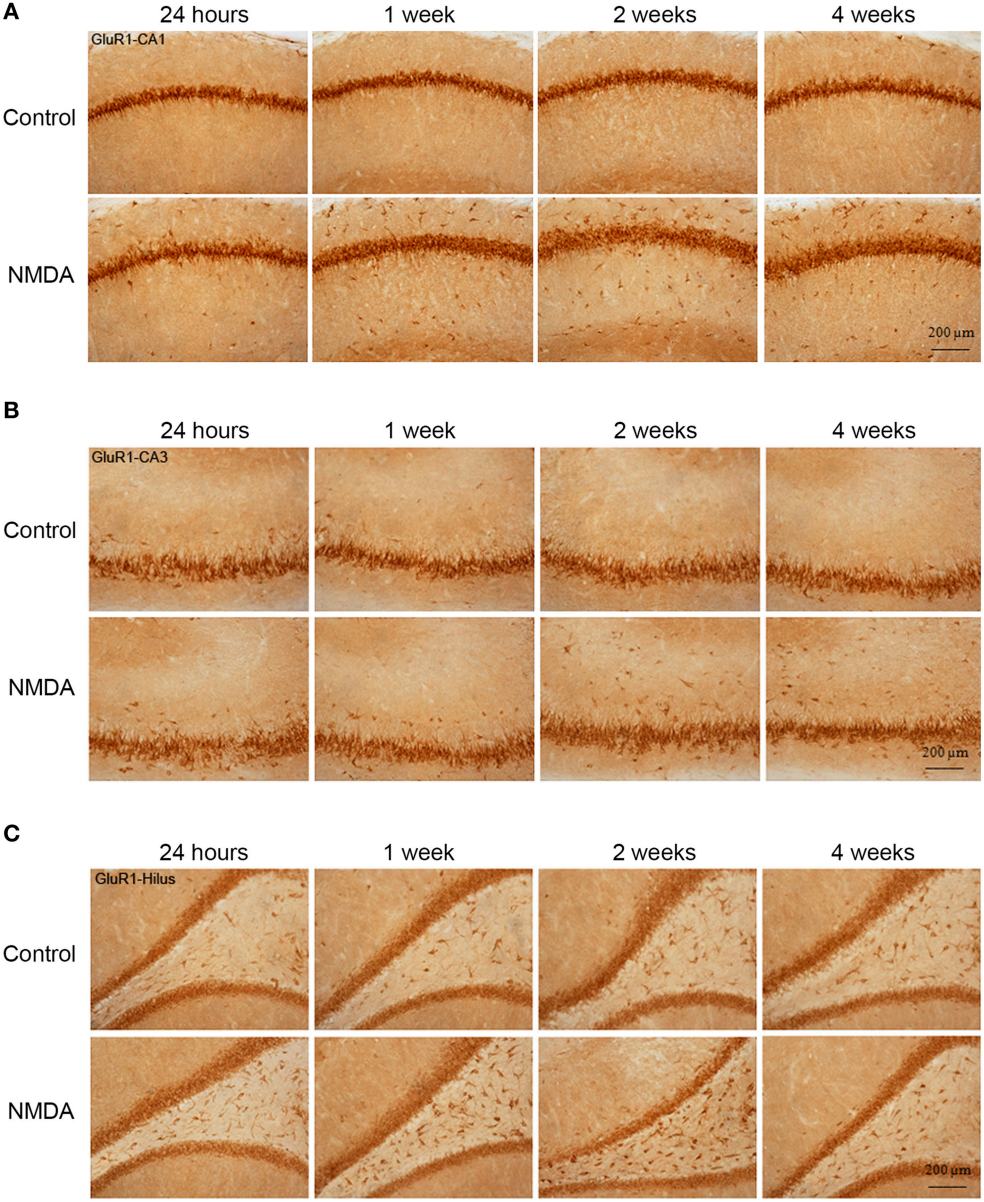
**Immunohistochemistry for GluR1 in CA1 (A), CA3 (B), and hilus (C) of the control and NMDA groups evaluated at 24 h, 1, 2, and 4 weeks after intrahippocampal injection**. Cells labeled with the chromogen 3,3-diaminobenzidine (DAB).

GluR1-immunopositive cells (IC) were observed in all hippocampal areas of control and NMDA groups at all evaluated time points (Figure 3) The expression of GluR1 was concentrated in the neuron layers of CA1, CA3, and hilus as well as in sparsely distributed cells throughout these areas (Figure 3). Specifically in CA1 area, the population of GluR1-IC in the control group remains as an organized layer over the evaluated period (Figure 3A). In the NMDA groups, GluR1-IC were observed both in the layer as well as randomly distributed around it. Additionally, the increased thickness of the cell layer detected in NMDA groups suggests a disruption in the region adjacent to the injury. In CA3 area, a higher number of GluR1-IC scattered around the dense layer of GluR1-IC was observed in NMDA compared with control groups at all evaluated time points (Figure 3B). Similarly, a higher density of GluR1-IC was observed in the hilus of the NMDA groups (Figure 3C).

**Figure 4:**
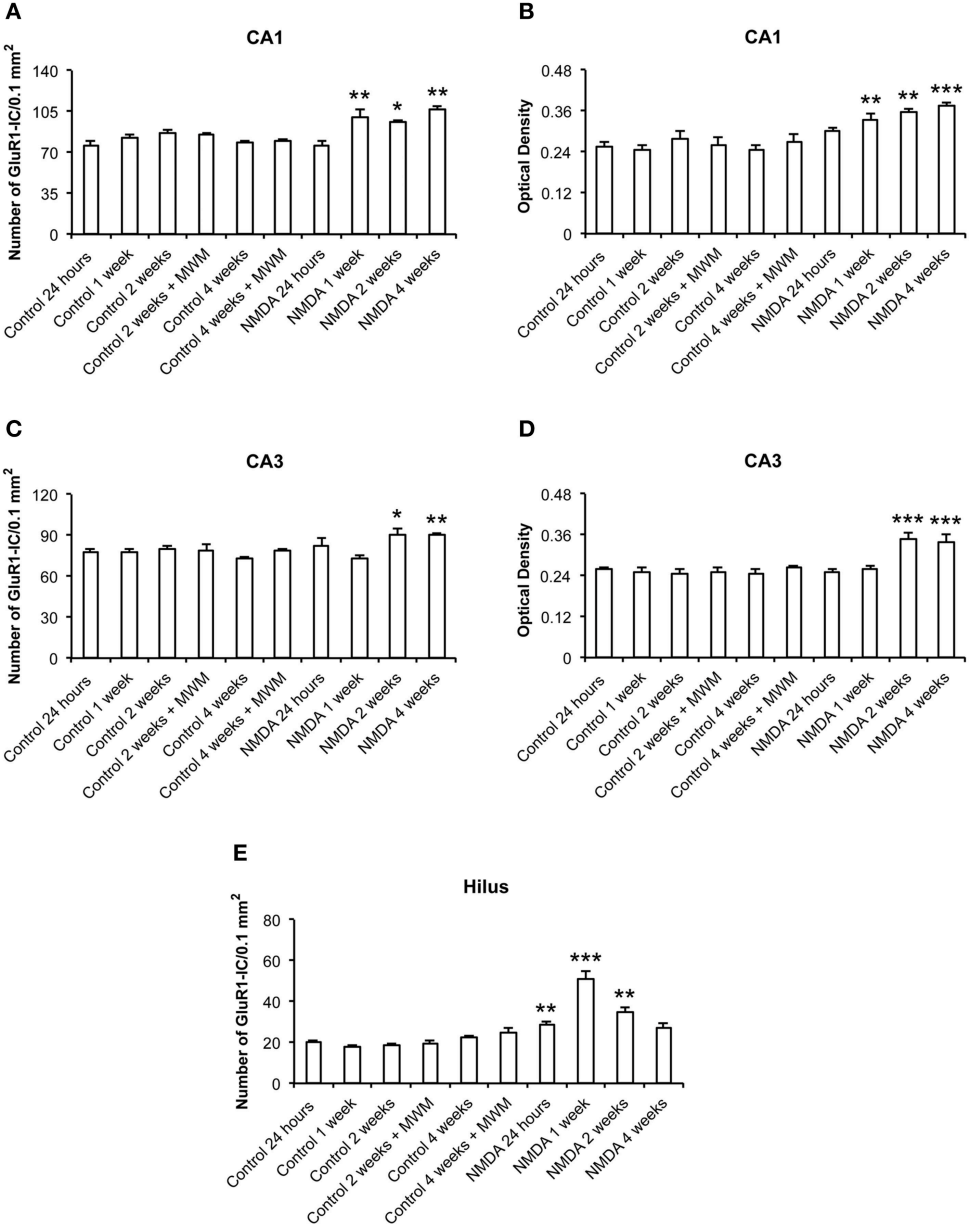
**Number of GluR1-immunopositive cells (A,C,E) and optical density (B,D) in CA1, CA3, and hilus of the control and NMDA groups evaluated at 24 h, 1, 2, and 4 weeks after intrahippocampal injection**. Data represent mean ± SEM of the number of cells and optical density quantified bilaterally in three sections/rat (*n* = 6/group). ^*^p < 0.05; ^**^p < 0.01; ^***^p < 0.001 (one-way ANOVA followed by Newman–Keuls test).

The quantitative analysis showed differences in GluR1 expression between control and NMDA groups regarding hippocampal area and time after injection (Figure 4). In CA1, the number of GluR1-IC increased in NMDA compared with control groups at 1 (33%), 2 (20%), and 4 (35%) weeks after injection [*F*_(9, 53)_ = 10.40; *p* < 0.0001] but did not change at 24 h (Figure 4A). The optical density exhibited the same pattern of GluR1-IC increasing in CA1 area in NMDA compared with control groups at 1 (34%), 2 (43%), and 4 (51%) weeks after injection [*F*_(9, 48)_ = 12.75; *p* < 0.001] without changing at 24 h (Figure 4B). In CA3 area, the number of GluR1-IC increased in NMDA compared with control groups at 2 (22%) and 4 (21%) weeks after injection [*F*_(9, 53)_ = 3.726; *p* < 0.01] but did not change at 24 h and 1 week (Figure 4C). The optical density exhibited the same pattern of GluR1-IC increasing in CA3 area in NMDA compared with control groups at 2 (37%) and 4 (37%) weeks after injection [*F*_(9, 49)_ = 6.640; *p* < 0.001] without changing at 24 h and 1 week (Figure 4D).

In the hilus, the number of GluR1-IC increased in NMDA compared with control groups at 24 h (41%), 1 (190%), and 2 (90%) weeks after injection [*F*_(9, 53)_ = 27.15; *p* < 0.0001] but did not change at 4 weeks (Figure 4E). The optical density was not measured in the hilus as the GluR1-IC were spread preventing the counting of individual cells. In addition, the pattern of cell distribution could lead to optical density values with considerable gray levels from the background area. No differences in GluR1-IC between the control groups either submitted or not to MWM behavioral test were observed at 2 and 4 weeks after injection in all evaluated areas (Figures 4A–E). Omission of the primary antibody anti-GluR1 resulted in complete loss of signal (data not shown).

### GluR2 expression in hippocampus

**Figure 5:**
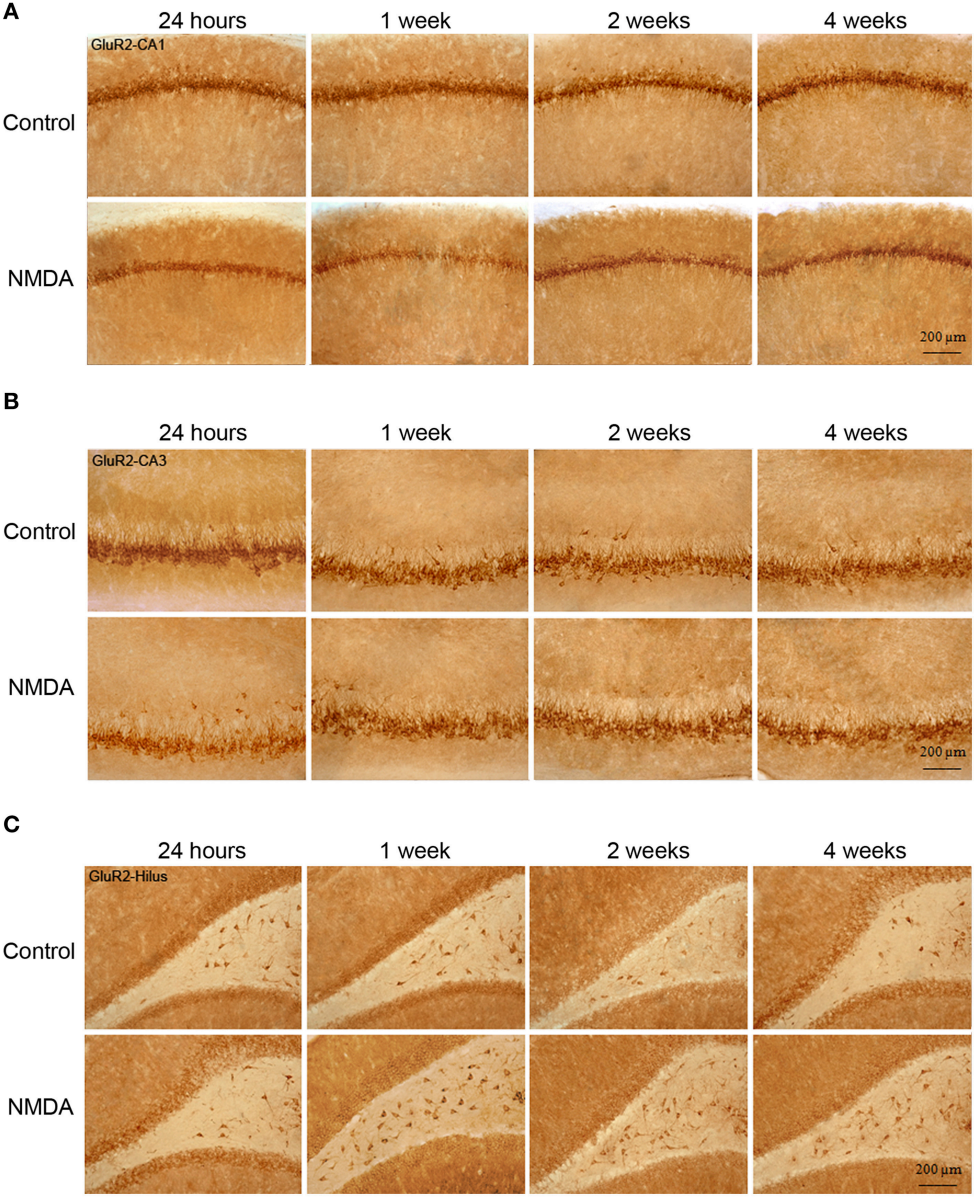
**Immunohistochemistry for GluR2 in CA1 (A), CA3 (B), and hilus (C) of the control and NMDA groups evaluated at 24 h, 1, 2, and 4 weeks after intrahippocampal injection**. Cells labeled with the chromogen 3,3-diaminobenzidine (DAB).

Similarly to GluR1, GluR2 expression was observed in all hippocampal areas of control and NMDA groups at all evaluated time points (Figure 5). However, GluR2-IC exhibited a different pattern of localization compared with GluR1. They were concentrated in cell layers and not sparsely distributed, except in CA3 where few GluR2-IC were noticed around the cell layer (Figure 5). In CA1, it was observed a reduction of the GluR2-IC layer thickness in NMDA compared with control groups at all evaluated time points (Figure 5A). In CA3, GluR2-IC was distributed in a very similar way in control and NMDA groups at all evaluated time points (Figure 5B). An increase of GluR2-IC was observed in the hilus of NMDA compared with control groups at all evaluated time points (Figure 5C).

**Figure 6:**
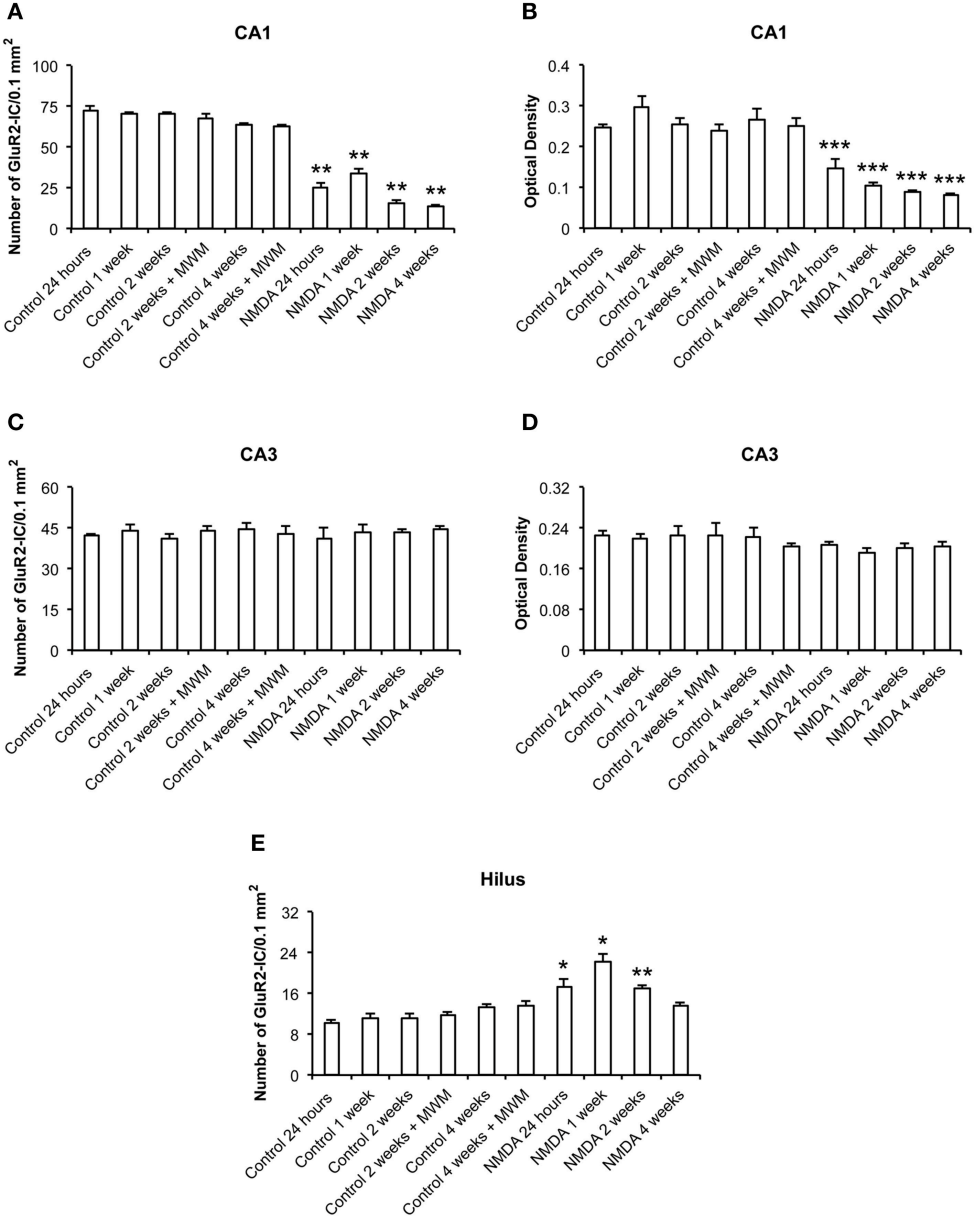
**Number of GluR2-immunopositive cells (A,C,E) and optical density (B,D) in CA1, CA3, and hilus of the control and NMDA groups evaluated at 24 h, 1, 2, and 4 weeks after intrahippocampal injection**. Data represent mean ± SEM of the number of cells and optical density quantified bilaterally in three sections/rat (*n* = 6/group). ^*^p < 0.05; ^**^p < 0.01; ^***^p < 0.001 (one-way ANOVA followed by Newman–Keuls test).

The quantitative analysis showed differences in GluR2 expression between control and NMDA groups in CA1 and hilus over the time after injection, but not in CA3 (Figure 6). In CA1, the number of GluR2-IC decreased in NMDA compared with control groups at 24 h (60%), 1 (53%), 2 (75%), and 4 (78%) weeks after injection [*F*_(9, 56)_ = 146.9; *p* < 0.0001] (Figure 6A). The optical density exhibited a similar pattern of GluR2-IC decreasing in CA1 area in NMDA compared with control groups at 24 h (44%), 1 (59%), 2 (65%), and 4 (69%) weeks after injection [*F*_(9, 49)_ = 23.50; *p* < 0.001; Figure 6B]. In CA3, it was not observed differences between NMDA and control groups regarding GluR2-IC amount as evaluated by both cell counting (Figure 6C) [*F*_(9, 53)_ = 0.3251; *p* = 0.96] and optical density (Figure 6D) [*F*_(9, 49)_ = 0.8004; *p* = 0.6181]. In contrast to the CA1, in the hilus the number of GluR2-IC increased in NMDA compared with control groups at 24 h (28%), 1 (65%), and 2 (25%) weeks after injection [*F*_(9, 53)_ = 18.18; *p* < 0.0001] but did not change at 4 weeks (Figure 6E). The optical density was not measured in the hilus as the GluR2-IC displayed the same pattern of distribution as described for GluR1 in this area. No differences in GluR2-IC between the control groups either submitted or not to MWM behavioral test were observed at 2 and 4 weeks after injection in all evaluated areas (Figures 6A–E). Omission of the primary antibody anti-GluR2 resulted in complete loss of signal (data not shown).

### GFAP and NeuN expression in hippocampus

**Figure 7:**
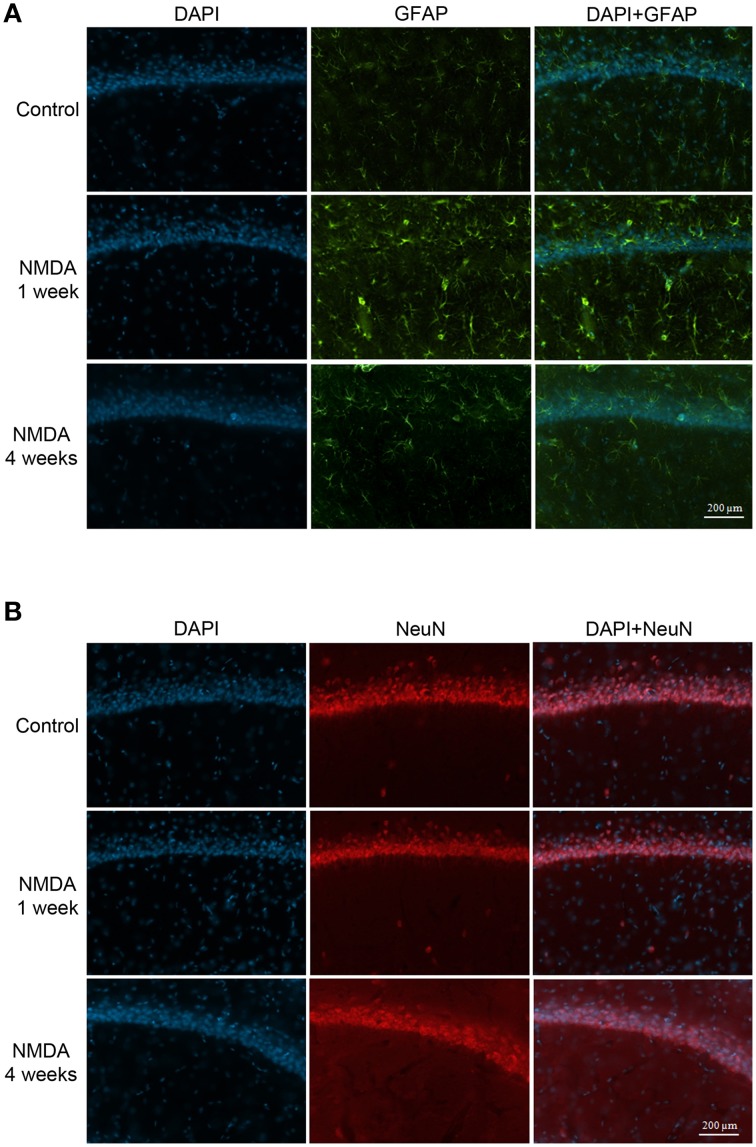
**Immunofluorescent labeling for glial cells (A, GFAP–green) and neuronal cells (B, NeuN–red) in CA1 (adjacent area of injection) of the control and NMDA groups 1 and 4 weeks after injection**. Nuclei labeled with DAPI (blue).

**Figure 8:**
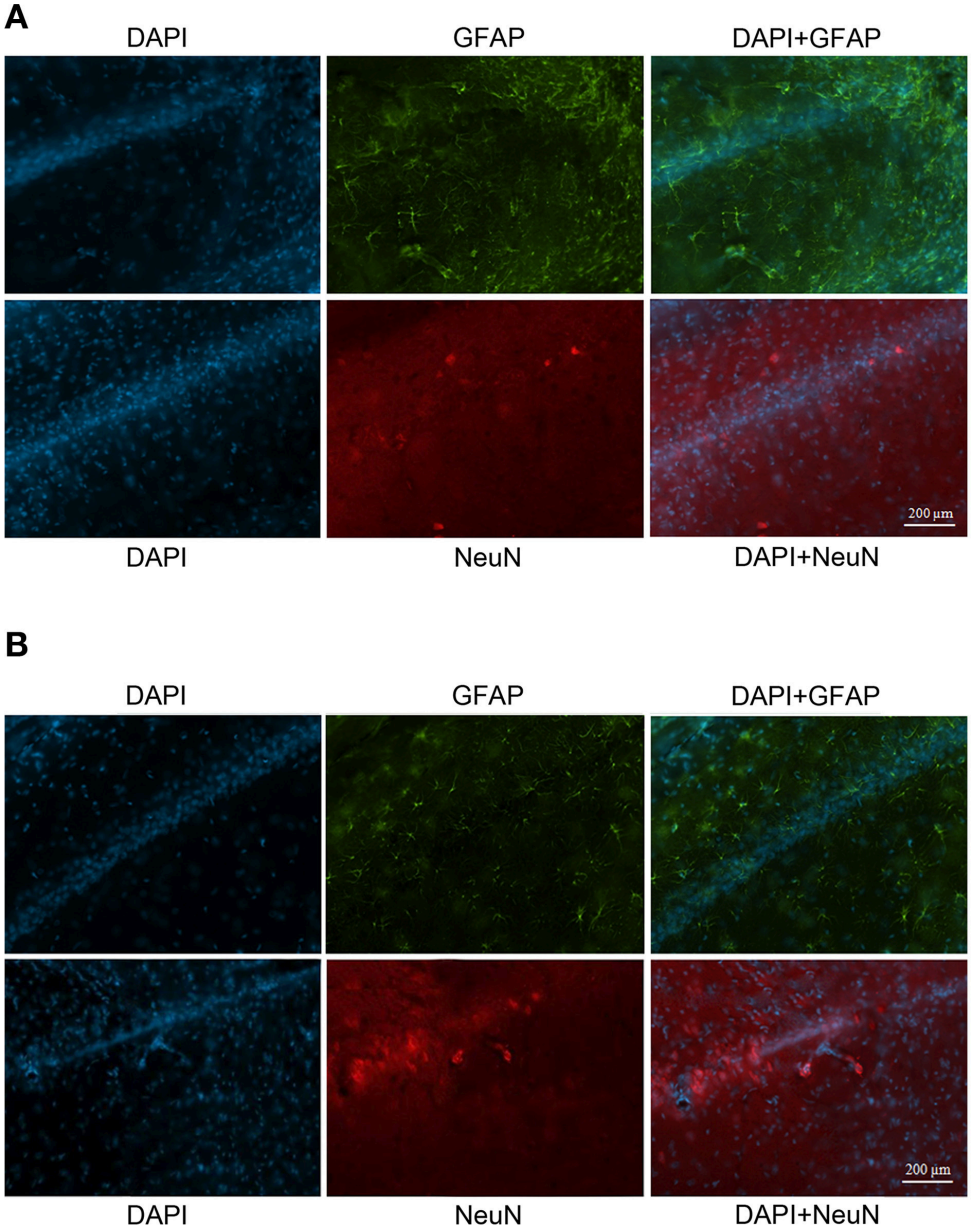
**Immunofluorescent labeling for glial cells (GFAP–green) and neuronal cells (NeuN–red) in CA1 (injected area) 1 week (A) and 4 weeks (B) after injection**. Nuclei labeled with DAPI (blue).

The GFAP labeling (green) was observed in control and NMDA groups in the adjacent area of injection 1 and 4 weeks after injection and it was associated with nuclei labeled with DAPI (blue; Figure 7A). As expected, the expression of GFAP increased in NMDA compared with control group 1 week after injection and reduced 4 weeks after injection to similar levels of control group. The NeuN (red) was also detected in all evaluated groups associated with cell nuclei (Figure 7B). In contrast with GFAP, the expression of NeuN was slightly reduced in NMDA compared with control group 1 week after injection and increased 4 weeks after injection reaching similar levels of control group. The expression of GFAP in the NMDA injected area 1 week after injection indicates the intense and disorganized glial proliferation induced by injury (Figure 8A). In addition, the absence of NeuN labeled cells indicates that NMDA injection induced nearly 100% of neuronal death in this area. As in these areas the nuclei may not be associated with neurons, they probably refer to glial cells. Four weeks after NMDA injection, GFAP expression was reduced (Figure 8B) compared with 1 week. In contrast with 1 week, NeuN expression was observed in this area 4 weeks after NMDA injection. The NeuN expression was mainly associated with the organized layer of nuclei in the image center. Panoramic views of the GFAP and NeuN labeling in the hippocampus of control rats are showed in the Supplementary Material (Figure S3).

## Discussion

The present study showed the time course of changes in the expression of GluR1 and GluR2 in hippocampus induced by a local injection of NMDA. Briefly, our results demonstrated that NMDA induced distinct alterations in the expression of each subunit depending on the specific hippocampus subfields and the time after injection.

The efficacy of the hippocampal lesion was analyzed in terms of function by the MWM behavioral test for learning and memory, and histologically by Nissl staining. The findings from MWM suggest that NMDA induced a partial deficit in the hippocampal functions as we observed impairment in memory evocation in all periods after injection, but not a complete deficit of learning. Although the animals injected with NMDA showed higher escape latency than controls to reach the platform when tested 2 and 4 weeks after injection, they learned the task, as the latencies progressively decreased over the four training days. In addition, in the *probe trial* without platform, even the NMDA groups spent considerable less time than controls in the target quadrant, staying there 30% of the total time. It has been reported that learning and memory functions essentially depend of the hippocampus integrity (Broadbent et al., 2004). These authors have shown that spatial memory is not completely disrupted by lesions affecting up to 40–60% of the total hippocampus and that lesions from 30 to 50% induce learning disabilities, but the animals do not stop learning. Furthermore, the application of colchicine into the rat dentate gyrus interferes with the spatial memory when tested in the MWM, radial, and T maze, while lesions only in CA1 did not tend to produce deficits (Costa et al., 2005). Therefore, the DG integrity is considered essential for spatial orientation. Both studies support our results as in our model of injury, although the histological findings from Nissl staining showed evident cell loss in CA1 and CA3, it was not detected in hilus, suggesting its integrity. These findings may explain the partial learning of animals injected with NMDA. Thus, the histological and functional efficacies of the hippocampal injury induced by NMDA were demonstrated in this study. This experimental design can also be used as a model of excitotoxic injury in further investigations to improve the understanding of neurodegenerative processes and the events arising from them.

As previously mentioned, it is well-known that different subtypes of glutamate receptors are largely distributed in CNS (Hollmann and Heinemann, 1994; Ozawa et al., 1998). Regarding the AMPA receptors, the distribution of distinct subunits is different among brain areas (Eastwood et al., 1994; García-Ladona et al., 1994) and neuronal types (Jonas et al., 1994). In this study, the expression of GluR1 and GluR2 was not uniform in hippocampus, in agreement with the classic distribution of AMPA receptors reported for the rat brain (Ozawa et al., 1998; Danbolt, 2001). In addition, it is known that the assembly of different subunits results in AMPA channels with different Ca^2+^ permeability (Mosbacher et al., 1994). Thus, the GluR1:GluR2 ratio changes the Ca^2+^ influx in specific cell populations and brain conditions such as stage of development, physiological functions, pathological processes, toxicicity, and neurodegeneration and mechanisms of plasticity (Hume et al., 1991; Pellegrini-Giampietro, 2003).

In this work, the hippocampal injury induced by NMDA resulted in a time course of distinct changes in GluR1 and GluR2 expression. Considering CA1, the injected hippocampal subfield, the GluR1 expression in cells adjacent to the lesion was increased, while the expression of GluR2 was decreased during the whole time course evaluated. These alterations result in an increase in GluR1:GluR2 ratio with consequent excessive Ca^2+^ influx in this subfield that may promote neurotoxic processes. Our histological results showed death cells in CA1 1 week but not 24 h after NMDA injection (results from 24 h are not shown), consistent with the increased expression of GluR1, the AMPA subunit highly permeable to Ca^2+^, at 1 week but not at 24 h. It is probable that the mechanisms of neurotoxicity had been immediately triggered after NMDA injection, but they did not result in changes in the expression of GluR1 detected by our method. However, GluR2 expression was highly decreased 24 h after NMDA injection, suggesting that a reduced GluR2 role in regulating Ca^2+^ influx through AMPA receptors is part of the mechanisms of toxicity firstly triggered by NMDA that results in neuronal death later.

Considering the time course evaluated in this study, the changes in the expression of both subunits were observed up to 4 weeks after NMDA injection. While NMDA immediately elicits toxicity, the resulting neurodegeneration triggers plasticity mechanisms in order to keep the functional integrity of the brain. Glutamate, through distinct receptors, has been shown to underlie the brain excitability in response to high influx of Ca^2+^ that is crucial for cell proliferation, neurogenesis and synaptogenesis, among other mechanisms of neuronal plasticity and regeneration (Molnar et al., 2002; Rakhade et al., 2008). The involvement of AMPA receptors in mediating fast synaptic transmission has been demonstrated to contribute to synaptic plasticity that underlies learning and memory (Du et al., 2004; Fei et al., 2006; Harrison et al., 2007; Zhang et al., 2007). Malinow (2003) showed that the presence of GluR1 subunit is linked to the LTP formation that is essential for strengthening and effectiveness of synapses in both during the postnatal development and the recovery after brain injury. Additionally, it was demonstrated that GluR1 subunit plays a critical role in mediating synaptic plasticity and cognition (Malinow and Malenka, 2002; Suk et al., 2004). Based in all these evidences, our results suggest that the changes in GluR1 and GluR2 expression until 4 weeks after NMDA injection may be involved in the mechanisms of plasticity triggered by the lesion.

The results from immunofluorescence showed complete absence of neurons labeled with NeuN in the lesioned area of CA1 at 1 week after NMDA injection, in agreement with our histological results using Nissl staining. We also observed intense labeling for GFAP in this area at the same time point evidencing the typical gliosis that occurs in response to neuronal insults. Agreeing with this, the upregulation of GFAP in astrocytes has been reported to be a spontaneous response to brain injury that contributes to both reactive gliosis and formation of the glial scarf (Sofroniew, 2009). In contrast, at 4 weeks after NMDA injection we observed few NeuN-lebeled neurons in the lesioned CA1 suggesting a possible initial reorganization of the area. Based on our data, it is not possible to confirm that these NeuN-positive cells are new neurons since a specific label for this kind of cells such as bromodeoxyuridine (BrdU) was not used. However, the absence of NeuN-positive cells 1 week after lesion suggests that the neurons in the lesioned area after 4 weeks were originated from migration of the DG subgranular zone. This hypothesis is supported by recent findings demonstrating an increase in glial cells and BrdU-positive granular neurons in the hippocampus of mouse after cerebral trauma (Gao and Chen, 2013). These authors suggest that the trauma triggers innate mechanisms of plasticity and repair through the proliferation of progenitor cells. In this context, previous experiments demonstrated that *in vivo* astrocytes from human lateral ventricle showed a behavior similar to cultured neural progenitor cells (Sanai et al., 2004).

In a different context, Ming and Song (2005) reported that specific kinds of injuries may activate neurogenesis through endogenous neural progenitor cells in areas where the adult neurogenesis is limited. However, the authors do not known if these new neurons are functional. So, these findings also contribute to explain our results from the memory test on the MWM 4 weeks after NMDA injection when the animals remained with cognitive deficit.

It has been reported an increase of neurogenesis in hippocampus of animals submitted to MWM or physical exercises related to learning (Epp et al., 2011; Inostroza et al., 2011; Chow et al., 2012). In addition, Huang et al. (2010) reported an increase in the expression of GluR1 in CA1 of rats with impaired fetal growth after being exposed to MWM. Together, these findings show that the exposure to specific physical conditions may improve learning and memory functions and that GluR1 is involved in these mechanisms (Huang et al., 2010). In agreement with this, Okada et al. (2003) showed that an increase of Ca^2+^ influx in CA1 through AMPA receptors is associated with learning. In this context, the present work shows that only the exposition to MWM was not enough to activate the subunits GluR1 and GluR2, as we did not observed significant differences in the expression of both subunits when the animals treated with saline and exposed to MWM were compared to the animals also treated with saline but not exposed to MWM. Such finding suggests that only the exposition to behavioral test do not trigger mechanisms of plasticity and repair. However, considering that the mechanisms of plasticity were triggered by an excitotoxic stimulus, the MWM test becomes important as it involves physical exercise and learning and memory processes. Altogether, changes in the expression of GluR1, GluR2, GFAP, and NeuN in the lesioned CA1 over 4 weeks after NMDA injection suggest that AMPA subunits are involved in distinct mechanisms activated in a time-dependent way, i.e.,: excitotoxicity that lead to cell death at the initial periods after injury and plasticity events at the later evaluated time point.

The time course of the changes induced by NMDA injection in the expression of GluR1 and GluR2 in CA3 and hilus exhibited different patterns. While in CA3 we observed an increase in GluR1 without alteration in GluR2, in the hilus both subunits were increased at all evaluated time points. Considering that the subgranular zone of the dentate gyrus is rich in stem cells and the subfields of the dentate gyrus have distinct cells types, our findings may be related to either the appearance of new cells or changes in the assembly of AMPA receptor subunits, agreeing with the plasticity mechanisms triggered in response to injury. Zhang et al. (2011) showed that the dentate gyrus acts as a filter to block the exacerbation of signal from cortex to CA3 and then to CA1, suggesting that any neuronal lost in dentate gyrus is able to disturb the normal balance between excitation and inhibition in this area.

It has been demonstrated that the electrical stimuli in dentate gyrus are controlled by different types of GABAergic interneurons that play a crucial role in the control of hippocampus-cortex interactions and in maintaining the dentate gyrus integrity (Zhang et al., 2011). Thus, the neuronal loss in the hilus could induce a decrease in the inhibition by GABA resulting in an increase of the dentate gyrus excitability. In addition, these interneurons express calcium-binding proteins that have a role of buffering the calcium excess (do Nascimento et al., 2012). These findings contribute to explain the differential susceptibility of each area to degeneration, as the neurons with few or none calcium-binding proteins are more sensitive to death triggered by excitotoxicity. They also corroborate the low susceptibility of dentate gyrus neurons to death by excitotoxicity observed in this study as CA1, but not dentate gyrus, was the site of NMDA injection in our model of injury. Furthermore, the expression of AMPA subunits observed in the hilus 1 and 2 weeks after injury exhibited an increased GluR1:GluR2 ratio, indicating more vulnerability to death at this time points. In contrast, similar increase in the expression of both subunits was observed 4 weeks after injury, resulting in lack of change in GluR1:GluR2 ratio suggesting that this specific assembly of subunits in the receptor underlies the mechanisms of maintenance of the dentate gyrus integrity after the excitotoxic stimulus. Conversely, in CA3 an increase only in GluR1 was observed 2 and 4 weeks after injury, increasing GluR1:GluR2 ratio and contributing to the vulnerability of this subfield to neurotoxic mechanisms. These findings are similar to those reported by Jiang et al. (2008) after induction of seizures in hippocampal cells cultures.

The brain mechanisms underlying the injury, plasticity or repair triggered in response to NMDA intrahippocampal injection are extremely complex and involve other cerebral areas and neurotransmitters than hippocampus and glutamate (Bowie, 2008). This study demonstrated a distinct balance between GluR1 and GluR2 along the time course evaluated. In addition, both subunits have two isoforms, flip and flop, with distinctive kinetic properties that may influence Ca^2+^ influx depending on the assembly of subunits in the receptor, as the flip isoform allows greater influx of this ion (Sommer et al., 1990).

Considering the glutamatergic loop in the hippocampus (Bloodgood and Sabatini, 2008) that involves synaptic connections among hilus, CA3, and CA1 together with all evidence described above, it is possible that the distinct changes in GluR1 and GluR2 expressions are related to the complex glutamatergic mechanisms triggered in hippocampus in response to NMDA injection resulting in a local injury and in the activation of neuronal plasticity.

## Author contributions

HF, AP, MI, and MR. All authors had substantial contributions to the conception or design of the work; or the acquisition, analysis, or interpretation of data for the work; and drafting the work or revising it critically for important intellectual content; and final approval of the version to be published; and agreement to be accountable for all aspects of the work in ensuring that questions related to the accuracy or integrity of any part of the work are appropriately investigated and resolved.

### Conflict of interest statement

The authors declare that the research was conducted in the absence of any commercial or financial relationships that could be construed as a potential conflict of interest.
