# Supplementary material for: Differential Expression of AMPA Subunits Induced by NMDA Intrahippocampal Injection in Rats
Source: Front Neurosci. 2016 Feb 15;10:32. doi: 10.3389/fnins.2016.00032 (PMC4753315; doi:10.3389/fnins.2016.00032)
Supplement: Supplementary file 1 [file Image1.PDF]

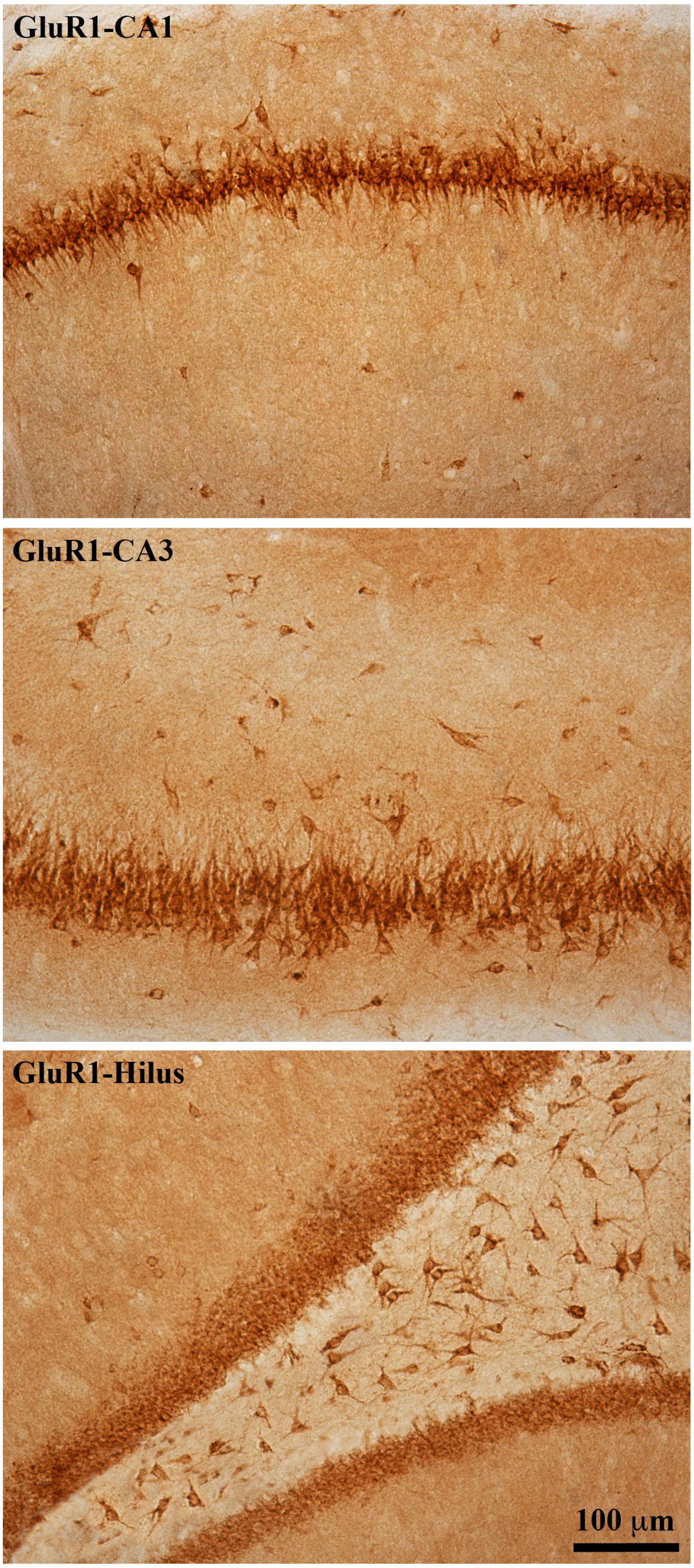

Figure S1. Immunohistochemistry for GluR1 in CA1, CA3 and hilus in higher magnification. Cells labeled with the chromogen 3,3-diaminobenzidine (DAB). Images used for quantifying the positive cells.
