# Supplementary material for: Differential Expression of AMPA Subunits Induced by NMDA Intrahippocampal Injection in Rats
Source: Front Neurosci. 2016 Feb 15;10:32. doi: 10.3389/fnins.2016.00032 (PMC4753315; doi:10.3389/fnins.2016.00032)
Supplement: Supplementary file 3 [file Image3.PDF]

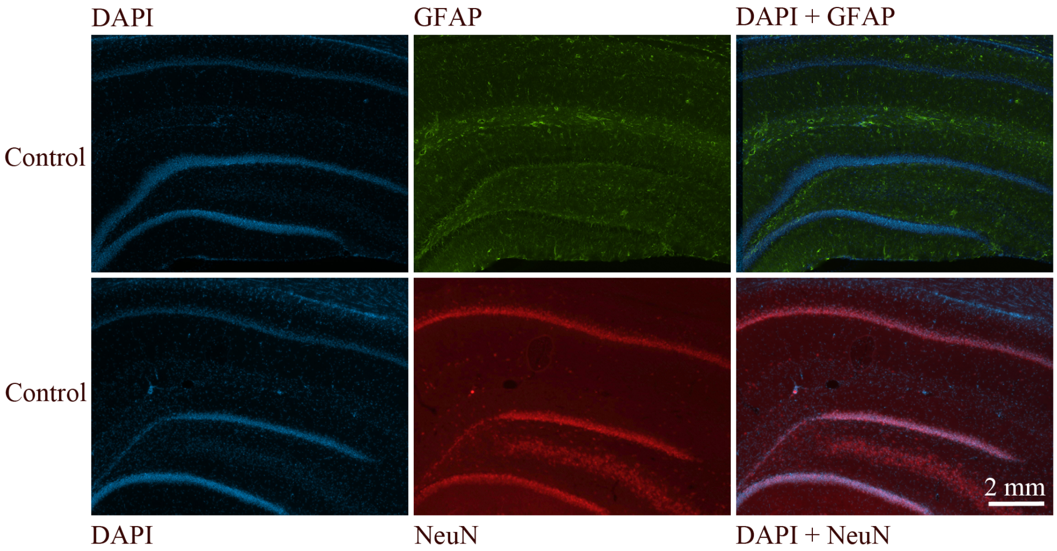


**Figure S3.** Immunofluorescent labeling for glial cells (GFAP - green) and neuronal cells (NeuN - red) in the hippocampus of control rats. Nuclei labeled with DAPI (blue).
